# Trypanosomes in Neotropical frogs: unveiling hidden megadiversity and complex host–parasite patterns

**DOI:** 10.1098/rsob.250190

**Published:** 2025-11-19

**Authors:** Jan Votýpka, Miloslav Jirků, Viktoria Spodareva, Jana Režnarová, Klára Poloprutská, Petr Pajer, David Milner, Thomas Richards, Vyacheslav Yurchenko, Roberto Ibáñez, Julius Lukeš, Alexei Yu. Kostygov

**Affiliations:** ^1^ Department of Parasitology, Faculty of Science, Charles University, Prague, Czech Republic; ^2^ Institute of Parasitology, Biology Centre, Czech Academy of Sciences, České Budějovice, Czech Republic; ^3^ Zoological Institute, Russian Academy of Sciences, Saint Petersburg, Russia; ^4^ Life Science Research Centre, Faculty of Science, University of Ostrava, Ostrava, Czech Republic; ^5^ Military Health Institute, Military Medical Agency, Prague, Czech Republic; ^6^ Department of Biology, University of Oxford, Oxford, UK; ^7^ Smithsonian Tropical Research Institute, Panama City, Panama; ^8^ Faculty of Science, University of South Bohemia, České Budějovice, Czech Republic

**Keywords:** Anura, Trypanosomatidae, mixed infections, evolution, diversity, ecology, conservation

## Abstract

Amphibia-infecting members of the genus *Trypanosoma* represent its most divergent lineage and exhibit remarkable morphological and genetic diversity. However, their detailed study has been hindered by pleomorphism, morphological convergence, multiple infections and scarcity of molecular data. By combining light microscopy with molecular phylogenetics, we investigated trypanosome diversity in a broad sample of frogs from Panama, a climatically stable tropical biodiversity hotspot. The uncovered diversity of trypanosomes parasitizing amphibians was exceptional, exceeding the host species richness twofold. Phylogenetic analyses revealed conspicuous ecological partitioning: distinct lineages were primarily associated with the arboreal hylids, with only rare host switches to understorey species, suggesting that vector feeding preferences structure the parasites’ community. Notably, none of the identified haplotypes matched those from South America, underscoring geographic isolation as a driver of diversification. This study revealed a vast, previously undescribed diversity of trypanosomes that reflects the ecological and taxonomic breadth of their amphibian hosts. Given the ongoing global amphibian decline, documenting these parasitic communities is urgent. Our findings highlight how host ecology and biogeography shape parasite evolution, and offer a framework for future research in threatened tropical ecosystems.

**Figure:**
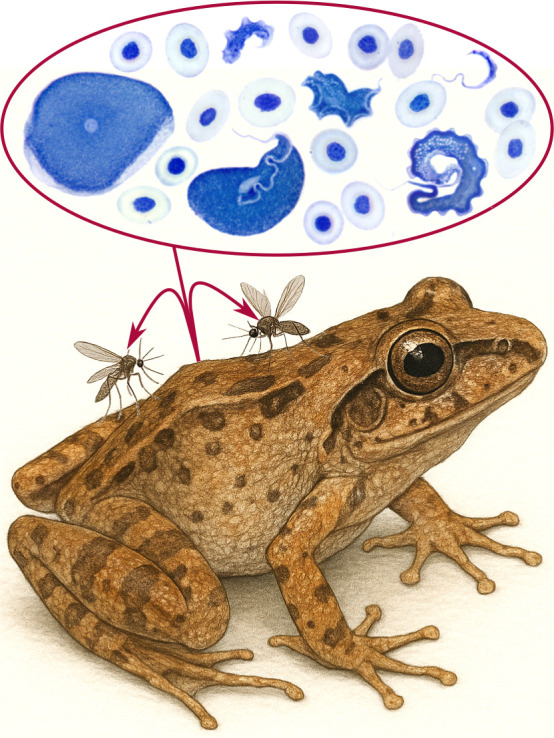

## 1. Introduction

The genus *Trypanosoma* comprises about 500 described species parasitizing all vertebrate classes and is best known for the members pathogenic to humans and domestic mammals [1]. This well-known taxon was originally established nearly two centuries ago for flagellates discovered in frog blood, and the first three (or four, depending on synonymy) species were described in anurans [2–4].

Amphibian trypanosomes exhibit greater morphological diversity than those infecting other vertebrates. Apart from the canonical fusiform trypomastigotes found across vertebrate hosts, the cells parasitizing the amphibian bloodstream can be rounded, oval, claviform, flabellate, foliaceous or irregularly shaped, with or without a free flagellum, and sometimes displaying longitudinal or spiral striations [5,6]. Although such diversity of shapes should aid species discrimination, several factors complicate its taxonomic utility: extensive intraspecific pleomorphism, morphological convergence among species, frequent co-infections and a critical lack of reference molecular data for most nominal taxa [7–10]. This makes the systematics of amphibian trypanosomes particularly difficult and contributes to frequent classification errors that further amplify taxonomic confusion.

Research on this group is often constrained by small sample sizes, narrow geographical scope and restricted host range. Due to these limitations, the known diversity of amphibian trypanosomes remains disproportionately low compared to that of their hosts. To date, only about 90 species and a comparable number of distinct 18S rRNA gene sequences have been described from nearly 9000 extant anuran species [11]. This disparity is further exacerbated by a general reluctance among researchers to establish new trypanosome species in this group, as it would require considerable effort to clarify taxonomic relationships. Consequently, a lack of taxonomic rigour has led to improbable records, such as the type species *Trypanosoma rotatorium* being reported from over 60 anuran species across at least five anuran families and four biogeographic realms: Afrotropical, Nearctic, Neotropical and Oriental [6,8,12–14]. Similar over-generalizations concern other amphibian trypanosome species, albeit with narrower host and geographical ranges [12,15,16]. Molecular phylogenetic analyses have revealed that trypanosomes bearing the same name may represent distinct, occasionally not even directly related, species [7].

From an evolutionary perspective, amphibian trypanosomes represent a key group for understanding the origin and radiation of the genus *Trypanosoma*. Early molecular phylogenies revealed a basal split between the ‘aquatic’ (anuran and piscine) and ‘terrestrial’ (reptilian, avian and mammalian) lineages [17,18]. Expanded taxon sampling not only supported this dichotomy but also demonstrated that amphibian trypanosomes form a vast and highly diverse group, paraphyletic with respect to trypanosomes from leech-transmitted non-amphibian aquatic hosts such as fish, platypus, turtles and crocodiles [7,8,19–22]. These findings suggest that trypanosomes colonized vertebrates through tetrapods, with amphibians—the earliest lineage—probably serving as their primary host group. While this hypothesis cannot be confirmed using existing data, the phylogenetic positions of lizard-infecting *Trypanosoma therezieni* and *T. tokoloshi* support the occurrence of host switches from amphibians to terrestrial amniotes during evolution [23].

Studies addressing amphibian trypanosome diversity using molecular data on representative datasets remain scarce, yet the available evidence already indicates high species richness. In the most extensive survey to date, conducted in Brazil, 29 trypanosome haplotypes (probably distinct species) were identified in 259 frog specimens representing 47 species from eight families across the Amazon, Atlantic Forest and Pantanal biomes [8]. Similarly, a study focused on just two members of the genus *Pelophylax* (out of the 25 currently recognized species, ranging from the Atlantic to the Pacific [24]) across several sites in Central and Eastern Europe documented five trypanosome species [7]. These examples illustrate that trypanosome diversity may rival that of their amphibian hosts.

In this study, we combined light microscopy and molecular phylogenetics to show that taxonomically and ecologically diverse amphibian assemblages in palaeoclimatically stable tropical regions host an unexpectedly high number of trypanosome species. Our findings further reveal how host ecology shapes the spatial distribution and diversity of these parasites.

## 2. Material and methods

### 2.1. Sample collection and establishment of cultures

In November 2018, 100 individuals representing 19 frog species (about 10% of the anuran diversity in Panama and <6% of that in Central America [25,26]) were collected in Central Panama. These species spanned 15 genera and 8 families (table 1; electronic supplementary material, table S1). Eighty specimens were gathered at altitudes of 30–200 m above sea level across six sites within Soberanía National Park, all located within a 10 km radius of the Gamboa research facility of the Smithsonian Tropical Research Institute. The remaining 20 frogs were sampled in pre-montane rainforests: 18 near El Valle de Antón (650 m) and two in Altos de Campana National Park (900 m) (electronic supplementary material, table S1).

**Table 1.** List of analysed anuran species and their trypanosome prevalence.

family	species	prevalence
Bufonidae	*Rhinella alata*	2/8 (25%)
	*R. horribilis*	2/6 (33%)
Centrolenidae	*Sachatamia albomaculata*	0/1 (0%)
Craugastoridae	*Craugastor crassidigitus*	0/3 (0%)
	*C. fitzingeri*	20/25 (80%)
Dendrobatidae	*Dendrobates auratus*	2/2 (100%)
Hylidae	*Agalychnis callydrias*	0/4 (0%)
	*Boana rufitela*	0/2 (0%)
	*Dendropsophus ebraccatus*	0/3 (0%)
	*Hypsiboas rosenbergi*	0/1 (0%)
	*Scinax boulengeri*	0/1 (0%)
	*S. ruber*	2/2 (100%)
	*Smilisca sila*	13/15 (87%)
	*Trachycephalus venulosus*	3/3 (100%)
Leptodactylidae	*Engystomops pustulosus*	0/2 (0%)
	*Leptodactylus insularum*	2/3 (67%)
	*L. savagei*	7/9^a^ (78%)
Ranidae	*Lithobates warszewitschii*	2/7 (29%)^b^
Strabomantidae	*Pristimantis gaigei*	1/3 (33%)
total		56/100 (56%)

^a^ For one of the positives, no sequence data could be obtained.

^b^ Parasites observed in fresh blood were not detected by PCR or stained smear analysis.

Blood samples were primarily obtained via cardiac puncture, after which the apparently healthy animals were released. Trypanosome presence was assessed by direct microscopic examination of fresh blood under a light microscope with a 40× objective. From each frog, one to three blood smears were prepared, air-dried, methanol-fixed and stained with Giemsa. For DNA extraction, one or two drops of blood were added to a solution of 2% SDS and 0.1 M EDTA, preserved at ambient temperature during fieldwork, and then transferred to the laboratory for storage at –20°C.

To establish cultures, several drops of blood were injected through a rubber cap into sterile 5 ml glass vials containing a biphasic medium. The solid phase consisted of 20% ovine blood agar, while the overlay comprised a 2 : 1 : 1 mixture of RPMI 1640, M199 and Schneider’s Insect Medium supplemented with 10% heat-inactivated foetal bovine serum, 500 U ml^−1^ penicillin, 100 μg ml^−1^ amikacin, 100 μg ml^−1^ streptomycin, 50 μg ml^−1^ chloramphenicol and 15 mg ml^−1^ 5-fluorocytosine (all from Sigma-Aldrich). Cultures were kept at room temperature, passaged 3–5 times in flat-sided tubes containing either biphasic or monophasic RPMI 1640 medium, and cryopreserved with 5% DMSO [27].

### 2.2. DNA isolation, PCR and sequencing

Total DNA was extracted from both preserved field samples and laboratory cultures using the DNeasy Blood & Tissue Kit (Qiagen) according to the manufacturer’s protocol. The resulting DNA was used to amplify the nearly full-length 18S rRNA gene (2.0–2.1 kb), using S762 and S763 primers for cultures, as described previously [28], or with TrN-F2 and TrN-R2 primers [29] for field-collected samples as specified below. A 25-μl PCR reaction contained 1 μl of template DNA, 1U of AccuTaq polymerase (Sigma-Aldrich), 1× LongAmp buffer, 500 μM dNTPs, 1M betaine, 1% DMSO and 400 nM of each primer. To minimise the formation of chimeric products, which commonly occurs when amplifying mixed templates, chaotropic additives (betaine and DMSO) were included, and the elongation step was performed at 65°C for 5 min [30].

Purified PCR products were used to prepare libraries using the Native Barcoding Expansion 96 kit and the Ligation Sequencing Kit (Oxford Nanopore). Sequencing was performed on the GridION platform using R9.4 chemistry. Base calling of raw reads was conducted using Guppy v. 5.1.13 [31].

### 2.3. Nanopore data processing

Sequencing reads were de-multiplexed, trimmed and, if internal adapter sequences were detected, split using Porechop v. 0.2.4 [32]. Species clusters and/or representative sequences were identified using NanoCLUST with the following parameters: -min_read_length 1800, -max_read_length 2100, -cluster_sel_epsilon 1 and the minimum cluster size threshold set to 4% of the total reads per sample. If the pipeline crashed, the values of cluster_sel_epsilon and the minimum cluster size were manually adjusted to achieve visually acceptable clustering results. Only clusters/sequences representing more than 4% of the total reads were retained for further analysis.

Relative abundance values of the identified species clusters were estimated using a custom R script. Sequences detected in trypanosome cultures but absent in the NanoCLUST output were searched for by lowering the cluster size threshold or directly among processed reads using BLASTn [33]. All obtained sequences were tested for chimeric artefacts using Bellerophon online software, with and without distance corrections (Jukes-Cantor, Kimura and Huber-Hugenholtz), across three sliding window sizes (200, 300 and 400) [34]. All identified haplotypes were deposited to GenBank under the accession numbers PV425878–PV425916 (electronic supplementary material, table S2).

### 2.4. Analysis of haplotype distribution across hosts

To assess whether the number of shared parasite haplotypes and haplogroups between host species exceeded random expectations, we conducted a permutation test in Python using the Pandas and NumPy libraries [35,36]. A null distribution of shared parasite richness was generated via 10 000 randomizations, in which parasite haplotypes/haplogroups were reassigned to the host species, while keeping the original number of haplotypes or haplogroups per host. The observed shared richness was then compared to this null distribution to calculate a one-tailed *p*‐value, with the test direction determined by the sign of the observed difference. Similarity networks illustrating the number of shared trypanosome haplotypes or haplogroups among host species were constructed and visualized using the Python packages NetworkX v. 3.4.2 and Matplotlib v. 3.9.3 (Pyplot module), respectively [37,38].

### 2.5. Phylogenetic analyses

Sequences of individual haplotypes (species) were combined with publicly available data from GenBank, including anuran trypanosomes (>1.4 kb long), representing aquatic trypanosomes from non-anuran hosts, and several species from terrestrial hosts used as outgroups. All sequences were aligned using MAFFT v. 7.490 with the E-INS-i algorithm [39]. The alignment was trimmed with trimAl v. 1.5 using a gap threshold of 0.5 [40]. A maximum likelihood phylogeny was reconstructed using IQ-TREE v. 2.3.6, employing automatic model selection and estimating branch support via 1000 standard bootstrap replicates [41]. Bayesian inference was performed in MrBayes v. 3.2.7 using the SYM+I+G model, sampling every hundredth generation across 2 000 000, with all other parameters set to default. Additional approximately 700 nt-long sequences of Neotropical anuran trypanosomes were retrieved from GenBank if they showed 100% identity to sequences already included in the dataset. Accession IDs, definitions and host or vector data for these sequences were added to those of their longer counterparts at the tree tip labels.

### 2.6. Morphological analysis

Giemsa-stained blood smears from all specimens positive for trypanosomes were examined under a light microscope using a 100× oil immersion objective. All trypanosome cells were photographed and assigned to morphotypes based on morphological features. Measurements were performed in Fiji v. 1.54j software [42]. Cell length was measured excluding the free flagellum, whereas cell width was defined as the maximum transverse distance, including any folds of the undulating membrane. The cell side bearing the undulating membrane was designated as dorsal, and the opposite one was referred to as ventral. Where possible, morphotypes were linked to 18S rRNA haplotypes based on co-occurrence, relative abundance and the assumption that morphologically similar trypanosomes may have related haplotypes.

## 3. Results and discussion

### 3.1. Parasite prevalence

Trypanosomes were detected in 56 out of 100 analysed anuran specimens either by PCR and/or by light microscopy (table 1; electronic supplementary material, table S1). As expected, PCR was the most sensitive method, identifying 53 positive samples compared to 39 detected by fresh blood microscopy and 49 by stained smears. Only one smear-positive sample failed to yield a PCR product, whereas five PCR-positive individuals showed no trypanosomes in the blood smears. These discrepancies probably reflect variation in parasitaemia: among positive slides, trypanosome counts ranged from 6 to 1500 cells. Notably, in two samples from Warszewitsch’s frog (*Lithobates warszewitschii*), trypanosomes were observed in fresh blood but could not be detected by PCR or microscopy of stained smears. The live trypomastigotes (observed by J.L. and M.J.) were small, shorter than erythrocytes and resembled insect-dwelling monoxenous trypanosomatids. We speculate that these flagellates might possess unusual features in the 18S rRNA gene that could interfere with primer binding or amplification.

Parasite prevalence among anuran species ranged from 0% to 100%, although these extremes should be interpreted with caution due to the small sample sizes. Among taxa with over five individuals examined, prevalence varied substantially: from 25% to 33% in *Rhinella* spp. or 29% in *L. warszewitschii*, to 78% in *Leptodactylus savagei*, 80% in *Craugastor fitzingeri* and 87% in *Smilisca sila* (table 1). If not due to sampling bias, the low prevalence in *Rhinella* spp. may reflect their thick, rigid and dry skin, reducing their attractiveness to dipteran vectors such as frog-biting midges (Corethrellidae). In the case of *R. horribilis*, a preference for artificial habitats might limit its contacts with forest-associated haematophagous insects. However, this explanation does not apply here, as all individuals of this species were collected at forest edges alongside heavily infected frogs of other taxa. In contrast, the high infection rates in *L. savagei*, *C. fitzingeri* and *S. sila* may be attributed to factors such as high population density, large body size and/or mating calls that are particularly appealing to dipteran vectors, either because of their intensity or synchronization [43–45].

### 3.2. Sequence analysis

A total of 183 sequences of the 18S rRNA gene obtained in this study clustered into 39 haplotypes, designated as PAF-00 to PAF-38 (electronic supplementary material, table S3). Each haplotype was detected in 1–15 individuals (median: 4). Pairwise sequence similarity ranged from 91.3% to 99.6% (electronic supplementary material, table S4). As there is no universally accepted sequence identity threshold for species delimitation based on the 18S rRNA gene sequences, we compared our data to reported values from mammalian trypanosomes. For instance, *T. melophagium* (HQ664912) and *T. trinaperronei* (MN752212)—two well-defined and closely related species of the subgenus *Megatrypanum* that differ in hosts, vectors and geographic range—share 99.6% identity [46]. Using this reference point, all haplotypes identified in this study were considered distinct species. Notably, none of the identified haplotypes exactly matched any sequences available in GenBank. However, two haplotypes exhibited high similarity to previously published sequences: PAF-20 shared 99.6% identity with EU021225, while PAF-26 shared 96.7% identity with EU021231.

Screening using Bellerophon with a 400-nt window revealed no evidence of chimeric sequences, regardless of distance correction. In contrast, runs using smaller window sizes (200 and 300 nt) combined with various correction parameters identified 15 putative chimaeras (electronic supplementary material, table S5). However, upon manual inspection, all these were deemed false positives, since they contained regions not matching the presumptive parents and displayed at least one additional line of evidence: (i) recurrence across multiple samples (identical artefacts are unlikely); (ii) detection by Sanger sequencing in monospecific cultures; (iii) occurrence as the sole haplotype in a sample; and (iv) absence of the candidate parental sequences in any sample from the same host (electronic supplementary material, table S5). Therefore, we concluded that none of the sequences obtained in this study was chimeric.

### 3.3. Morphology of trypanosomes in the bloodstream

Trypanosomes exhibit considerable size diversity. In most hosts, especially mammals, where they have been most extensively studied, these flagellates retain the classical fusiform trypomastigote morphology [47,48]. In contrast, in frogs, they display the greatest variation in cell shape, primarily reflected in markedly broader cell widths [5,49]. Indeed, the examined blood smears revealed striking morphological diversity, encompassing many previously described anuran trypanosome morphotypes as well as novel forms. However, classifying these forms, and especially correlating them with molecular haplotypes, was complicated by frequent coinfections (i.e. presence of multiple trypanosome species per sample), pronounced pleomorphism and morphological similarity among some taxa. Occasional artefacts from uneven drying and fixation, local cell aggregation, and other technical issues posed additional challenges. Furthermore, no morphological data were available for some species identified by sequences, due to the absence of trypanosomes in the blood smears. Conversely, in a few other cases, amplification and/or sequencing limitations prevented recovery of all trypanosome sequences present in a sample. The most prominent example was the sample PAH-01, which contained at least five distinct morphotypes but yielded only two haplotypes.

The encountered morphotypes are described below. For clarity, some were grouped based on their overall similarity. Due to the factors outlined above, the number of observed morphotypes exceeded that of the identified haplotypes. Consequently, a single species may exhibit more than one morphotype, but *vice versa,* a single morphotype may also correspond to multiple genospecies. Where feasible, correlations between morphotypes and haplotypes were proposed with varying levels of confidence, indicated as follows: possibly < presumably < likely.

#### 3.3.1. ‘*chattoni*-like’ morphotypes

**Figure 1.**
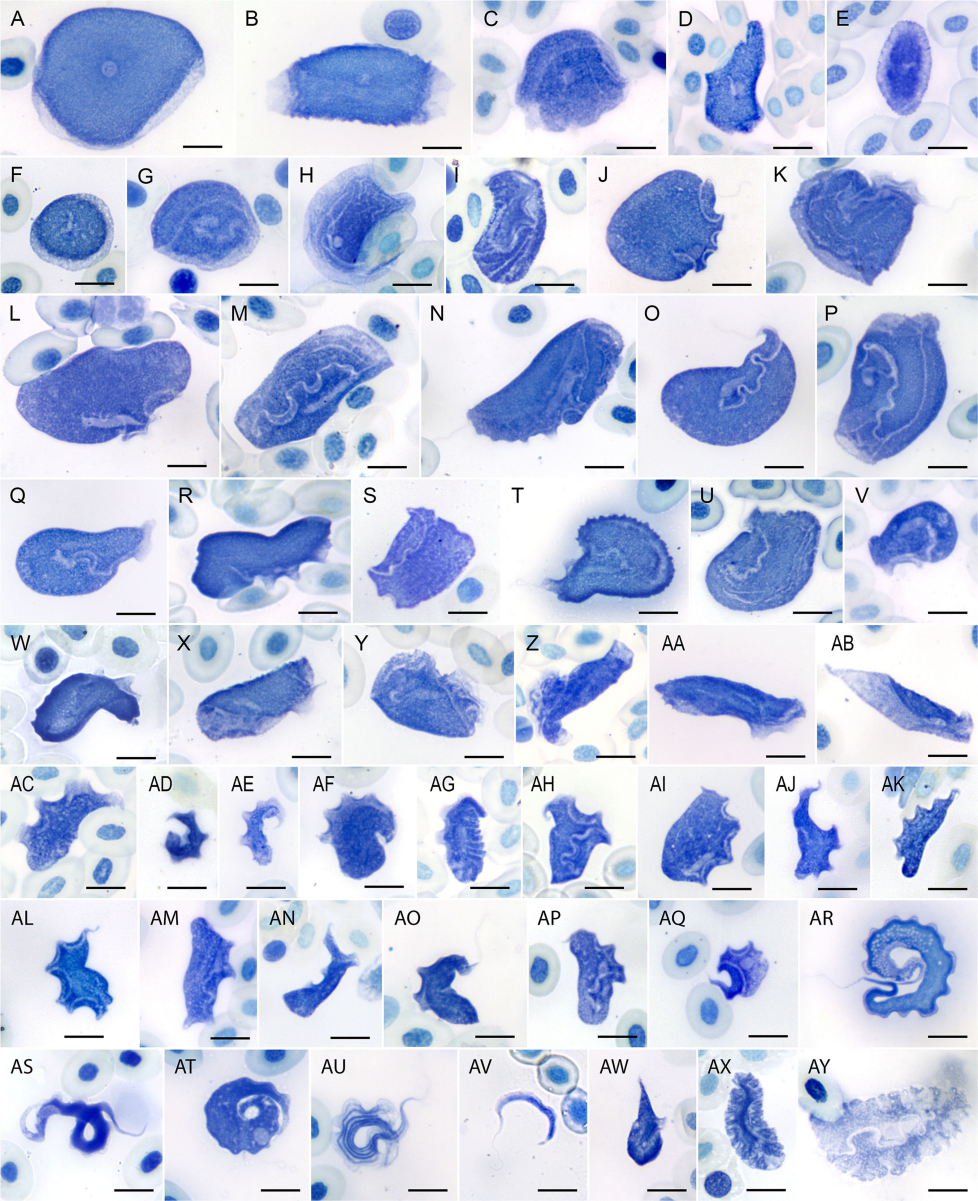
Morphotypes of trypanosomes in blood smears. Details of hosts (PAH-1–100) are provided in electronic supplementary material, table S1. (A–H) *chattoni*- and *tsunezomiyatai*-like; (I–Y) nautilus-like; (Z–AB) film-like; (AC–AP) *rotatorium*-like; (AQ–AT**,**AX,AY) ungrouped individual morphotypes**;** (AU–AW) snake-like. (A) ‘*chattoni*-like 1’, regular state, PAH-02; (B) ‘*chattoni*-like 1’, rolled and wrinkled state, PAH-54; (C) ‘*chattoni*-like 2’, PAH-92; (D) ‘*chattoni*-like 3’, PAH-16; (E) ‘flatbread’, PAH-01; (F) ‘saucer’, PAH-16; (G) ‘sesame’ PAH-21; (H) ‘kippah’, PAH-61; (I) ‘*nagasakiense*-like’, PAH-01; (J) PAH-53; (K) PAH-68; (L) PAH-53; (M) PAF-08; (N) PAH-60; (O) PAH-02; (P) PAH-68; (Q) PAH-53; (R) PAH-68; (S) PAH-94; (T) PAH-66; (U) PAH-55; (V) PAH92; (W) PAH20; (X) PAH-02; (Y) PAH-74; (Z) ‘crumpled’, PAH-01; (AA) ‘roll’, PAH-54; (AB) ‘sharptail’, PAH-60; (AC) PAH-01; (AD) PAH-88; (AE) ‘light’, PAH-02; (AF) PAH-70; (AG) ‘woodlouse’, PAH-01; (AH) PAH-91; (AI) PAH-86; (AJ) PAH-91; (AK) PAH-65; (AL) PAH-91; (AM) PAH-92; (AN) PAH-86; (AO) PAH-21; (AP) PAH-97; (AQ) ‘tailed’, PAH-97; (AR) ‘fishlet’, PAH-84; (AS) ‘polyclad’, contracted (natural?) state, PAH-60; (AT) ‘polyclad’, flattened state, PAH-60; (AU) ‘snake 1’, PAH-67; (AV) ‘snake 2’, PAH-28; (AW) ‘snake 3’, PAH-55; (AX) ‘pyjama’, PAH-16; (AY) ‘hook’, PAH-06. Scale bars = 10 μm.

Morphotypes in the ‘*chattoni*-like’ group (figure 1A–H) were compared to *T. chattoni*, originally described from the Vietnamese toad *Duttaphrynus melanostictus* [50]. The morphology of these parasites diverges farther from the prototypical ‘textbook’ trypanosome than any other known forms. The ‘*chattoni*-like 1’ morphotype (figure 1A), presumably corresponding to the haplotypes PAF-28, PAF-29 and PAF-31, was arguably the most prominent due to its large size (35–55 μm) and high abundance in some samples (over 200 cells per smear). These bowl-shaped cells exhibited various deformations affecting either the entire body or only its margin, such as flattening (giving them a pancake-like appearance), rolling (figure 1B), folding, corrugation, distortion, inflection and depression, resulting in a wide array of irregular shapes. The cytoplasm was homogeneous, with a transparent peripheral ring. The round nucleus (approx. 4  μm in diameter) was located centrally, and the small dot-like kinetoplast was positioned at its periphery. When visible, the flagellum was confined to a region surrounding the nucleus and measured only 1.7–3.5  μm in length. Trypanosomes with this morphology were found in the blood smears of *C. fitzingeri*, *Pristimantis gaigei* and *R. alata*.

Similar cells (morphotype ‘*chattoni*-like 2’), presumably corresponding to the related haplotype PAF-30 from *S. sila*, were smaller (approx. 30–35 μm in length), stained more intensely and exhibited little to no transparent peripheral area (figure 1C). A slightly different morphotype, ‘*chattoni*-like 3’ (possibly corresponding to haplotype PAF-32), was detected in the blood of *L. savagei* (figure 1D) and was represented by only a few irregularly shaped cells. They featured densely stained cytoplasm, lacked a discernible peripheral zone and contained a slightly elliptical nucleus larger than in other similar forms (approx. 5 × 4  μm), a prominent rod-like kinetoplast and a 4–6  μm long flagellum extending beyond the nuclear region. The overall size of these cells was difficult to estimate due to artefacts but appeared to fall within the range of the three aforementioned morphotypes.

The ‘flatbread’ morphotype (figure 1E) was distinct from members of the *chattoni*-like group, although it shared some morphological similarities with them. These cells were elliptical or oval, occasionally folded but never rolled, and measured 11–15 × 22–30 μm. They exhibited a dark-stained central cytoplasm typically surrounded by a lighter peripheral ring. The nearly central, round nucleus was approximately 3.5  μm in diameter, accompanied by a barely discernible kinetoplast located at the margin, associated with a bent, white structure 2.5–4  μm long. This structure was noticeably thicker than the flagellum seen in other trypanosomes and typically extended beyond the nuclear area. Cells of this morphotype were found in *Trachycephalus venulosus* PAH-01, where the number of distinct morphotypes exceeded that of haplotypes, preventing their confident correlation.

Since its description from *Glandirana rugosa* (Ranidae) and *Fejervarya limnocharis* (Dicroglossidae) in Japan, *T. tsunezomiyatai*, a species similar to *T. chattoni* but significantly smaller [51], has occasionally been used as a morphological reference. The ‘saucer’ morphotype found only in *L. savagei* PAH-16 and possibly corresponding to haplotype PAF-08 (figure 1F) most closely resembled *T. tsunezomiyatai*. These cells were flattened and slightly ellipsoid (22–26 × 18–20  μm), with a dark central cytoplasm surrounded by a lighter peripheral ring. The round nucleus (4–5 μm in diameter) was overlaid by a rod-like kinetoplast with a variable position. A thick flagellum (4–8 μm long) consistently extended beyond the nuclear area.

The ‘sesame’ morphotype (presumably corresponding to haplotypes PAF-13 and PAF-14) was represented by hemispherical cells 22–35  μm in diameter (figure 1G), with folding along the diameter being the most frequent deformation. The light peripheral margin was faint or indiscernible. The nucleus was sesame seed-shaped (3–4.5 × 5.5–7  μm), and a small, dot-like kinetoplast was positioned in close proximity to its posterior tip. Due to strong convolution, the thick 15–21 μm-long flagellum often passed over the nuclear region and typically terminated near the cell margin. This morphotype was observed in multiple samples of *C. fitzingeri*, as well as in *P. gaigei*, *R. alata* and *D. auratus*. It closely resembled *Trypanosoma bulat* described from *Limnonectes blythii* (Dicroglossidae) in Malaysia [52]. A similarly shaped morphotype, referred to as ‘kippah’ (figure 1H), featured slightly larger cells (29–41 μm) often bearing wrinkles. The nucleus was nearly round (3–4.5 × 2.5–4.0 μm), with a small, barely perceptible kinetoplast positioned adjacent to it. The short (5–8 μm) bent flagellum exhibited no consistent orientation. This morphotype, probably corresponding to haplotype PAF-27, was observed in a single specimen of *Trachycephalus venulosus*.

#### 3.3.2. ‘Nautilus-like’ morphotypes

A large and diverse group of forms, here collectively referred to as ‘nautilus-like’ (following a designation used for a previously identified morphotype in European green frogs [7]), were prevalent in the examined material (figure 1I–Y). While cells in this group varied substantially in size, proportions, overall shape, surface sculpture, nuclear dimensions, and the length of the undulating membrane, they shared the following characteristics: (i) a relatively broad cell body (at least three times wider than the undulating membrane), typically with a rounded posterior end; (ii) a blunt anterior end, occasionally with a sharply narrowed, claw-like extension; (iii) a well-defined nucleus (except in overstained cells); and (iv) from the origin of the undulating membrane to the posterior end comprising approximately 25% or more of the total cell length. The morphological diversity observed within this group exceeded the number of haplotypes that could be confidently associated with it, and in many cases, correlating sequences with specific morphotypes was not feasible.

The ‘*nagasakiense*-like’ morphotype, observed in *Trachycephalus venulosus* PAH-01, could not be reliably linked to a haplotype for the reasons outlined above. These slipper-shaped cells measured 13.5–18 × 30–44 μm and contained a large sausage-like nucleus (10–15 μm in length), with an undulating membrane extending along most of the cell body. This morphotype closely resembles *T. nagasakiense* described from *Hyla japonica* (Hylidae) in Japan [51] as well as trypanosomes detected in *L. chaquensis* in Brazil [53]. In other cases, considerable morphological variability was observed even when only a single haplotype was detected. For example, in *T. venulosus* PAH-74, which harboured only the unique haplotype PAF-37, cells varied in size (20–37 × 15–30 μm), exhibited a carrot-shaped nucleus (9–12 × 3–3.5 μm) and an undulating membrane spanning from two-thirds to nearly the entire cell length (figure 1Y).

#### 3.3.3. ‘Film-like’ morphotypes

A group of morphotypes represented by thin film-like cells (figure 1Z–AB) exhibiting various types of folding, rolling, and plication was observed across multiple samples, without any apparent phylogenetic or ecological association among them. The ‘crumpled’ morphotype (figure 1Z) was documented in the repeatedly referenced sample PAH-01, precluding its association with a specific haplotype. Cells of this morphotype appeared rolled into tubes of irregular diameter, with a funnel-like broadened anterior end bearing plications and/or vein-like structures bordered by an undulating membrane. Their fusiform nucleus, measuring 12–15 × 3–3.5 μm, was rarely discernible. These cells resembled an unnamed species previously reported from *Glandirana rugosa* (Ranidae) in Japan [51]. A similarly organised ‘roll’ morphotype (33–36 × 10–12 μm), lacking a conspicuous funnel, surface ornamentation, and visible nucleus, was observed in a single sample from *C. fitzingeri* (figure 1AA), yet no haplotype could be associated with it. The third, ‘sharptail’ morphotype, presumably corresponding to haplotype PAF-02, was the most loosely folded form, distinguished by a sharpened, typically unfolded and visually transparent posterior end (figure 1AB). The undulating membrane along one of its folded margins was generally inconspicuous. These cells resembled flagellates illustrated in the original description of *Trypanosoma borreli* from ‘*Hyla* related to *H. lateristriga*’ in Brazil [54]. Notably, this morphotype was detected in a single specimen of *S. ruber*, of which *H. lateristriga* is a junior synonym [55].

#### 3.3.4. ‘*rotatorium*-like’ morphotypes

Another diverse and prevalent group of morphotypes was referred to here as ‘*rotatorium*-like’ (figure 1AC–AQ), following a tradition established in the literature. These forms were generally smaller than the ‘nautilus-like’ morphotypes, with a broad (more than one-third of cell width), flounced undulating membrane and a body gradually tapering toward the anterior end. The nucleus was often indiscernible. Due to high variability, distinguishing discrete morphotypes or correlating forms across samples within this group was difficult; therefore, only selected examples are described below.

The ‘light’ morphotype (presumably corresponding to haplotypes PAF-15 and PAF-16) was characterized by a high contrast between the clearly visible, dense kinetoplast (located 2–5 μm from the posterior end) and a markedly transparent cytoplasm (figure 1AE). The cells measured 19–25 × 7–9 μm (including the undulating membrane) and exhibited a rounded posterior end. Due to a low contrast, the sausage-like nucleus was usually indistinct. When visible, the free flagellum measured 11–22 μm. Trypanosomes of this morphotype were found in *C. fitzingeri*, *L. insularum* and *S. sila*.

The ‘woodlouse’ morphotype (probably corresponding to either haplotype PAF-01 or PAF-35) featured cells measuring 20–30 × 7–13 μm, with pronounced wrinkling on the side opposite the undulating membrane and a rounded posterior end (figure 1AG). A well-developed undulating membrane extended along most of the cell length. The free flagellum, when visible, was approximately 8 μm long, and the elongated bullet- or sausage-like nucleus measured 9–10 × 2–3 μm. This morphotype was detected only in a single specimen of *T. venulosus*, in which it was predominant. A morphologically similar form has been previously reported from the same host species in Brazil [56].

The ‘tailed’ morphotype differed markedly from other *rotatorium*-like forms. It featured a narrow anterior end, contrasting with a significantly broader main body, and a drop- or lemon-shaped nucleus situated near the posterior end (figure 1AQ). A dot-like kinetoplast was positioned adjacent to a conspicuous indentation, representing the flagellar pocket, from which a flagellum approximately equal in length to the cell extended. This morphotype was recorded in four specimens of *S. sila*.

#### 3.3.5. ‘Snake-like’ morphotypes

Three morphotypes with a standard fusiform shape here collectively designated as the ‘snake-like’ group (figure 1AR–AT) featured long, thin and coiled cells with attenuated ends. They resembled *Trypanosoma tungarae* described from *Engystomops pustulosus* in Panama [57], as well as other species such as *T. neveulemairei* from Corsican *Pelophylax esculentus* [58] and *T. tsukamatoi* from Japanese *Limnonectes namiyei* [51].

The most prevalent member of this group, the ‘snake 1’ morphotype (figure 1AR), probably corresponding to the closely related haplotypes PAF-11 and PAF-12, was restricted to *C. fitzingeri*. Measuring 84–118 × 6–12  μm, these trypanosomes were the largest in our study. Highly convoluted cells appeared smaller and thinner, but their accurate measurements were not feasible. This morphotype featured fine longitudinal striations in the anterior half and numerous translucent vesicles in the posterior half of the cell. The flagellum emerged near the ellipsoid, obliquely oriented nucleus (4–5 × 2.5–3 μm), which was often indistinct due to a surrounding lightly stained zone extending both anteriorly and posteriorly along the ventral side.

The second largest ‘snake 2’ morphotype (65–91 × 4–9 μm) (figure 1AS), probably corresponding to haplotype PAF-26, was observed in *L. savagei*. These cells lacked visible striations or vesicles, and their cytoplasm stained uniformly dark. A pale perinuclear area extended anteriorly only and was separated from the posterior lighter region by a constriction of the dark cytoplasm.

Finally, the ‘snake 3’ morphotype (figure 1AT), presumably corresponding to haplotype PAF-00, was found in *L. insularum* and as a minor component in several specimens of *C. fitzingeri*. These cells were somewhat smaller (62–77 × 5–9.5 μm), had less attenuated ends than the preceding two forms and an 18–24 μm long flagellum. They lacked lightly stained cytoplasmic zones, striations or vesicles and were distinguished by the close proximity of the ovoid nucleus (4–6 × 3–5 μm) to the dorsal side of the cell.

#### 3.3.6. Other morphotypes

The ‘pyjama’ morphotype (figure 1AU), probably corresponding to haplotype PAF-09, was represented by elongated, coiled cells tapering anteriorly and broadening posteriorly, with a claw-like posterior end, from which a well-developed undulating membrane originated. Four to six (typically five) very prominent dark longitudinal streaks extended along the body, converging at the anterior end and fading towards the posterior. The diameter of the coils was 17–20 μm, and the free flagellum measured approximately 18 μm in length. The nucleus was not detectable. This morphotype was observed in several specimens of *L. savagei* and has been previously reported from *L. labyrinthicus* in Brazil [59].

The ‘hook’ morphotype, probably corresponding to haplotypes PAF33 and PAF34, consisted of long, slender cells (20–48 × 2–3.3 μm) tapered at both ends and typically curved like a hook or sickle; longer individuals often appeared coiled or S-shaped (figure 1AV). The kinetoplast was usually subterminal (1.7–2.5 μm from the rear tip), though in longer cells, stretching of the posterior part displaced it as far as 6–13 μm from the end. The oval nucleus, located centrally or in the anterior third of the cell, was often indistinct, whereas the narrow undulating membrane was well discernible, and the free flagellum varied in length (9–19 μm). Detected in *C. fitzingeri*, *L. savagei* and *S. ruber*, this morphotype closely resembles *T. ogawai*, originally described from the newt *Cynops ensicauda* in Japan [60].

An unusual ‘fishlet’ morphotype (figure 1AW) featured nearly drop-shaped cells (18–22 × 8–10.5 μm) with dense cytoplasm and a transparent undulating membrane. A rounded light structure (1.5–2  μm), likely a nucleus, was located centrally. Only a few such cells were observed in a single specimen (PAH-84), which harboured two non-unique haplotypes.

Two trypanosome cells from the blood smear of *S. ruber* PAH-60, each displaying a flexible ellipsoid body with plications on both sides, were tentatively assigned to a distinct ‘polyclad’ morphotype, named after the large marine flatworms. One of these cells (29 × 11 μm) appeared contracted and probably reflected a near-natural state (figure 1AX). The other cell (39 × 21 μm) was presumably artefactually flattened (figure 1AY), which nonetheless facilitated the observation of key internal structures positioned along the central axis: a wavy flagellum originating near a dot-like kinetoplast located 10 μm from the posterior end and a long, drop-shaped nucleus (4 × 12 μm). This morphotype possibly corresponded to the unique haplotype PAF-21.

### 3.4. Morphology of trypanosomes in culture

**Figure 2.**
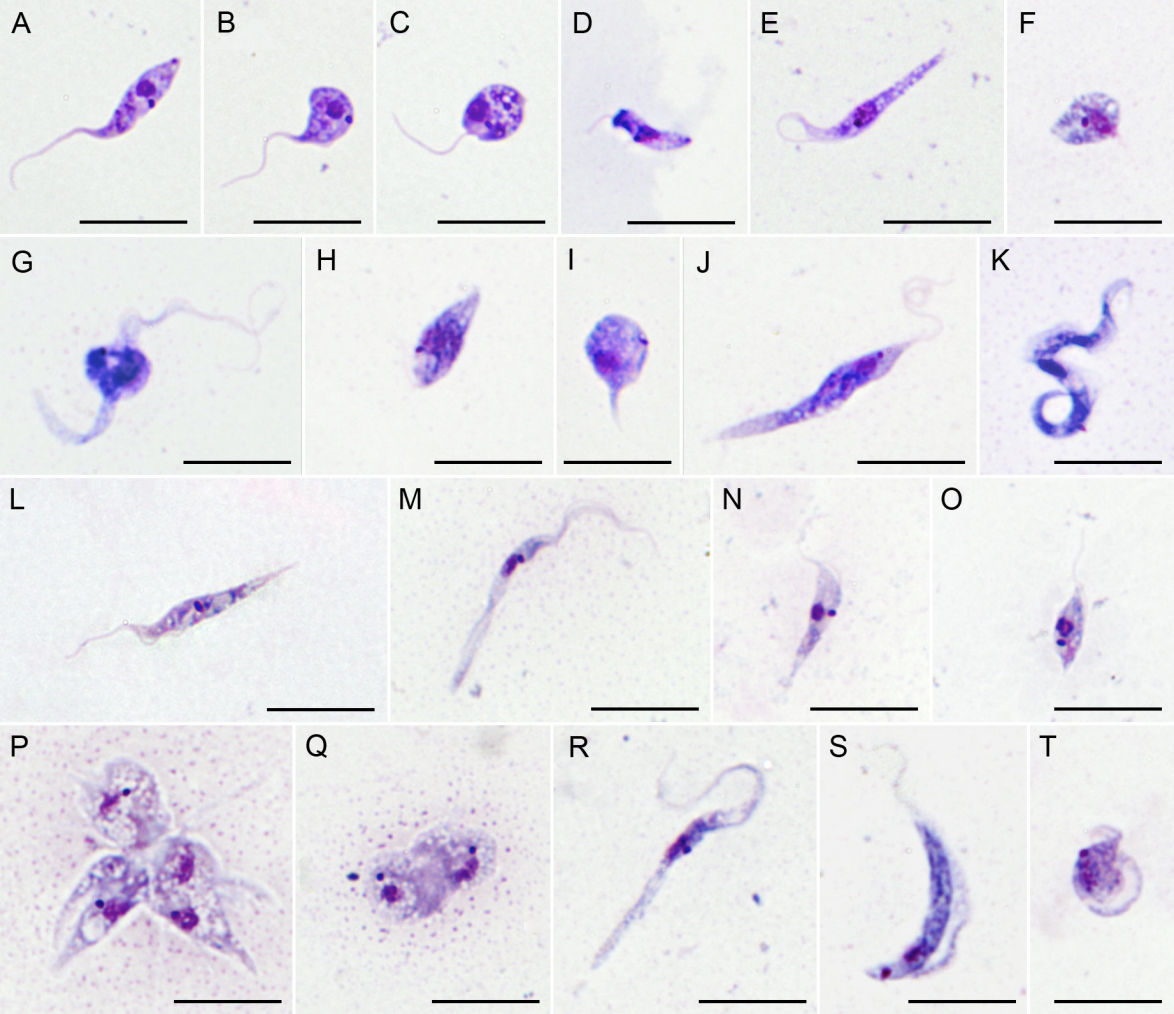
Morphology of trypanosomes in axenic cultures. Details of hosts (PAH-1–100) and haplotypes (PAF-00–38) are provided in electronic supplementary material, tables S1 and S3, respectively. (A–C) Haplotype PAF-00 (isolate PAH-56); (D–F) PAF-01 (PAH-01); (G–K) PAF-02 (PAH-60); (L) PAF-03 (PAH-61); (M–O) PAF-04 (PAH-92); (P, Q) PAF-05 (PAH-65). (R–T) PAF-06 (PAH-86). Scale bar = 20 μm.

Only a few trypanosomes documented in our study were successfully introduced into an axenic culture. Notably, none of the cultured cells exhibited the morphology observed in the bloodstream. Instead, they transformed into forms characteristic of both host-derived and cultured non-anuran trypanosomes (figure 2). This suggests that the cells undergo developmental changes during cultivation similar to those in the vectorial phase of the life cycle, which is consistent with previous observations in *Trypanosoma fallisi* and other species either maintained in culture or examined within their leech vectors [61,62].

The range of morphotypes observed and the details of the cell structure appear to be species-specific (figure 2A–T). In most cultures, epimastigote-shaped cells with a more or less developed undulating membrane predominated. These cells were elongated (figure 2A,E,L,M,N,R) or pyriform (figure 2B,T), with the kinetoplast located close to the nucleus, either anteriorly or laterally. Cells with a markedly swollen portion (figure 2G) were interpreted as intermediates in the transformation of elongated to pyriform epimastigotes. In one species (haplotype PAF-05), the culture consisted predominantly of rosettes containing elongated and/or rounded cells that lacked a visible flagellum (figure 2P,Q). Less frequent morphotypes included sphaeromastigotes (figure 2C,I) with anterior or posterior kinetoplasts, promastigote-like forms (i.e. more or less elongated cells with no discernible undulating membrane) (figure 2D,H,J), diverse trypomastigotes (figure 2K,O,S) and amastigotes (figure 2F).

### 3.5. Phylogenetic analysis

**Figure 3.**
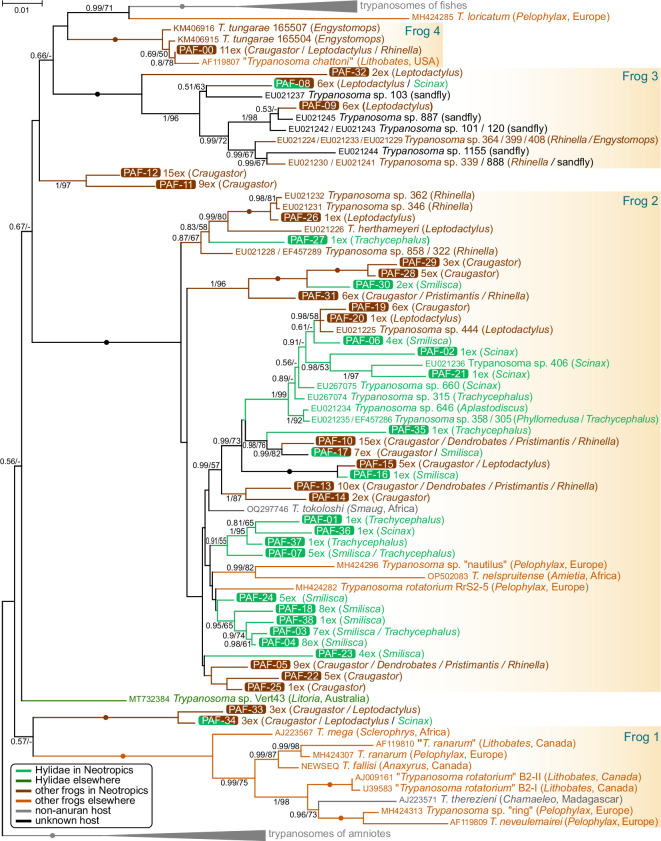
Maximum likelihood tree of ‘aquatic’ trypanosomes based on 18S rRNA gene sequences. The clades of trypanosomes parasitizing terrestrial vertebrates (used as an outgroup) and fishes (which also includes parasites of other aquatic animals such as turtles, crocodiles and the platypus) are collapsed. Taxa and tree branches are colour-coded according to the host groups (see graphical legend). The text in parentheses provides information about the host(s) or vector and geographic origin (for locations outside the Neotropics only). Bayesian posterior probability and bootstrap support values are indicated at branches for values ≥0.5 and ≥50, respectively. Maximal support by both methods (1.0/100) is denoted by black circles. The scale bar represents the number of substitutions per site.

The phylogenetic tree, inferred from the 18S rRNA sequences generated in this study and those retrieved from GenBank (figure 3), strongly supported the previously established clades ‘Frog 1’ through ‘Frog 4’ as well as the paraphyly of anuran trypanosomes with respect to those infecting fishes [7,57]. The Panamanian haplotypes were distributed among three of these clades but were notably absent from ‘Frog 1’, which so far lacks any sequences from the Neotropics. This distribution suggests that the divergence of the main clades probably occurred in the Early Mesozoic, prior to the breakup of the Pangaea supercontinent. Such a timeframe corresponds well with the early diversification of Anura in the Triassic to Early Jurassic [63]. This finding is particularly significant given that molecular data are currently available only for trypanosomes infecting Neobatrachia, a lineage that emerged in the Late Jurassic [63].

The vast majority of trypanosomes identified in this study (31 out of 39 haplotypes; 128 of 183 sequences) clustered within ‘Frog 2’, the largest clade in the tree, which encompassed over 60% (45 of 73) of all anuran trypanosome species analysed. Two pairs of Panamanian haplotypes formed distinct lineages outside the major clades, although their precise positions could not be confidently resolved due to low statistical support. Apart from these two small clades, no other lineages consisted exclusively of Neotropical isolates. Notably, the newly identified haplotypes from Panamanian frogs accounted for more than half (39 of 72) of all trypanosome species in the anuran section of the phylogenetic tree (figure 3).

Further analysis of the obtained sequences revealed a clear and ecologically informative pattern. First, of all the Panamanian haplotypes, 17 were found exclusively in arboreal treefrogs of the family Hylidae, while 19 were restricted to anurans of other families inhabiting the forest understorey (figure 3). Only three haplotypes were detected in both ecological categories, each consistently associated with either *Craugastor* or *Leptodactylus* species. The closest relatives of these three haplotypes were detected in Neotropical non-hylid frogs, suggesting a host-switching direction from the understorey to the arboreal frogs. Second, most trypanosome sequences from Hylidae clustered within three subclades of the ‘Frog 2’ clade. Two of these subclades were monophyletic and contained only Panamanian haplotypes, whereas the third was paraphyletic, with a small terminal group of trypanosomes from understorey frogs (*Craugastor* and *Leptodactylus*) and several isolates from Brazilian hylid hosts (figure 1). This pattern indicates that trypanosomes infecting treefrogs tend to remain specific to this ecological group and rarely switch to other anurans. Nevertheless, the observed distribution does not imply host–parasite coevolution, as no congruence was found between the phylogenies of treefrogs and their trypanosomes. Moreover, some trypanosome species within these lineages lacked host specificity, even at the genus level.

The ecological traits of Hylidae, and perhaps other canopy-dwelling anurans, probably account for the distinct composition of their trypanosome fauna. As their name implies, treefrogs are predominantly arboreal, whereas the other anuran families sampled in this study are largely confined to the forest understorey [26]. The observed differences in trypanosome species composition probably reflect adaptations of the parasites to the invertebrate vectors specialized for either canopy or understorey habitats. However, this ecological disjunction is not absolute, and occasional trypanosome host switching between these two frog categories does occur. Despite their primarily arboreal lifestyle, hylids descend to the understorey to breed [26,64]. Conversely, the green and black poison frog *Dendrobatus auratus*, although considered an understorey species, displays semi-arboreal habits and frequently ascends to trees. Moreover, its tadpoles develop in small ephemeral water bodies such as bromeliad leaf axils or tree holes. Consequently, all treefrogs are regularly exposed to the parasite pool of the understorey, whereas the presence of non-hylid anurans in the canopy is rather an exception. This ecological asymmetry explains the major direction of sporadic host switches—from the understorey to the arboreal frogs.

**Figure 4.**
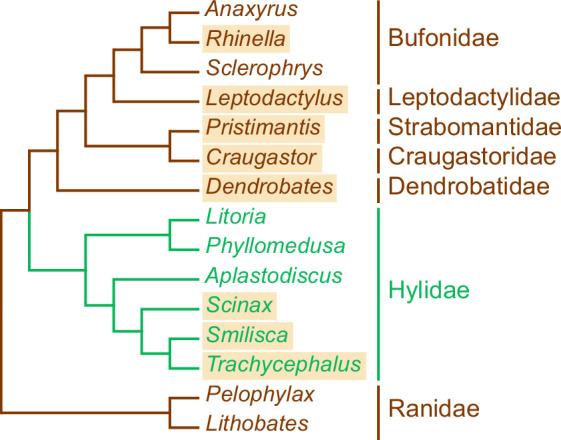
Cladogram depicting phylogenetic relationships among frog taxa. Members of the family Hylidae are shown in green; all other families are in brown. Taxa sampled in this study are highlighted. The cladogram was compiled based on previously published phylogenetic reconstructions, at the genus level for Bufonidae [65] and Hylidae [66], and at the family level for other taxa [63,67,68].

It is worth noting that among the trypanosome-infected Panamanian anurans examined here, Hylidae represent the most phylogenetically divergent family (figure 4). Therefore, they may possess distinct physiological traits that limit the compatibility of their trypanosomes with other frog taxa. However, host phylogeny does not appear to play a major role in shaping the trypanosome species composition (see below). Remarkably, the only available sequence of a trypanosome infecting a non-Neotropical treefrog [69] does not cluster within any of the established clades, suggesting yet another independent transition from understorey frogs to Hylidae.

### 3.6. Diversity patterns

Of the 39 haplotypes identified in this study, 27 (69%) were restricted to a single frog species, two were shared exclusively by the treefrogs *S. sila* and *T. venulosus*, and the remaining 10 were found in hosts from at least two different families (table 1; electronic supplementary material, table S6).

**Figure 5.**
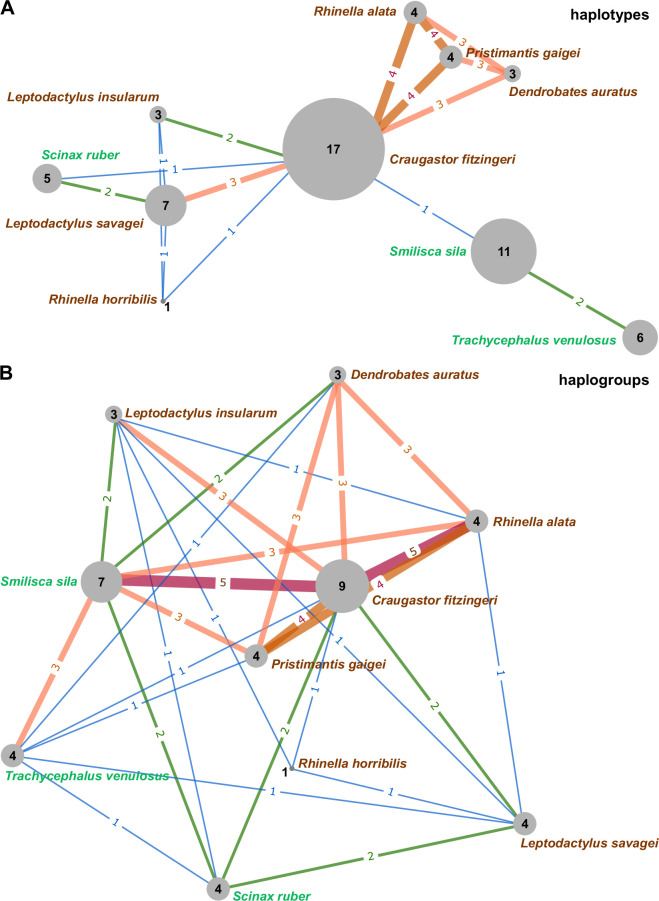
Similarity networks illustrating the sharing of trypanosome haplotypes and haplogroups among the studied anuran species. Nodes represent species, and edges indicate shared haplotypes (A) or haplogroups (B), highlighting patterns of genetic overlap. The number of shared haplotypes or haplogroups is represented by the colour and thickness of the edges, with exact numbers overlaid.

To gain a deeper insight into trypanosome distribution across Panamanian frogs, we summarized the presence of individual haplotypes in each host species and assigned them to haplogroups based on their phylogenetic relatedness (electronic supplementary material, tables S6 and S7). This dataset also served as a basis for a similarity network, illustrating the extent of haplotype and haplogroup sharing among host species (figure 5). Analysis of these distributions not only highlighted the strikingly limited overlap in parasite composition between the arboreal and the understorey frogs—previously observed in the phylogenetic tree—but also revealed notable species-level differences among anuran hosts.

*C. fitzingeri* stood out as the host species with the highest number of trypanosome haplotypes (17) and haplogroups (9), followed by the treefrog *S. sila* (11 and 7, respectively) (electronic supplementary material, tables S6 and S7). These counts strongly correlated with the number of infected individuals sampled per species, as confirmed by a significant positive relationship across the dataset (electronic supplementary material, figure S1). Given this, *C. fitzingeri* and *S. sila* were expected to share the highest number of trypanosome haplotypes. However, the observed overlap was significantly lower than predicted (*p* < 0.01; electronic supplementary material, table S8), indicating a pronounced spatiotemporal niche and vector separation between these frogs. Interestingly, this difference was not evident at the haplogroup level (electronic supplementary material, table S9), suggesting that the divergence of trypanosome assemblages infecting them is evolutionarily recent.

The extent of haplotype sharing with other frog taxa also differed substantially between *C. fitzingeri* and *S. sila*. The former harboured trypanosomes associated with both the arboreal and understorey species and occupied a central position in the haplotype-sharing network (figure 5A). Such a position, which persisted—though less prominently—at the haplogroup level (figure 5B), probably reflects the high abundance and year-round breeding of *C. fitzingeri* (electronic supplementary material, table S10), which may facilitate parasite exchange with other frogs. In contrast, *S. sila* was located at the periphery of the haplotype network, connected only to related *Trachycephalus venulosus* and haplotype-rich *C. fitzingeri*, but not to another treefrog, *Scinax ruber* (figure 5A). This pattern is consistent with the previously noted partial isolation of Hylidae-associated trypanosomes.

Interestingly, although *S. ruber* harboured fewer than half as many haplotypes as *S. sila*, both exhibited the same number of connections within the haplotype network (figure 5), suggesting that the latter species may possess unique traits limiting transmission of its trypanosomes to other frogs. Indeed, it was the only species in our dataset whose reproduction is associated with streams (electronic supplementary material, table S10), indicating potential involvement of distinct vectors. At the haplogroup level, *S. sila* showed a substantially higher number of connections, comparable to that of *C. fitzingeri* (figure 5B), indicating a relatively recent evolutionary divergence of its trypanosome fauna.

Both networks revealed a tight association of *C. fitzingeri* with a strongly interconnected cluster comprising *R. alata*, *P. gaigei* and *D. auratus* (figure 5), a pattern further supported by a statistically significant excess of shared trypanosome haplotypes and haplogroups compared to random expectations (electronic supplementary material, tables S8 and S9). These three hosts exhibited nearly identical parasite compositions but did not share any trypanosomes with either of the two *Leptodactylus* species. This is particularly notable, as *Leptodactylus* is the closest relative of *Rhinella* included in our dataset (figure 4), and both genera co-occurred syntopically (electronic supplementary material, table S1). The observed pattern suggests that *R. alata, P. gaigei* and *D. auratus* share ecological and/or physiological traits that set them apart from *Leptodactylus*.

Compared to members of this cluster, *L. savagei*, the third-ranked species by number of positive individuals and identified trypanosome haplotypes, exhibited only a weak association with *C. fitzingeri*, especially at the haplogroup level (figure 5; electronic supplementary material, tables S8 and S9). However, the observed number of shared haplotypes between these two species did not deviate from random expectations (electronic supplementary material, table S8), indicating no strong ecological overlap or separation. Two haplotypes were shared, one of which was also present in the treefrog *S. ruber*, further indicating some permeability between the arboreal and the understorey host ecological groups.

Notably, *L. insularum*, the closest relative of *L. savagei*, shared with it only one haplotype, PAF-00, which was detected in the culture from the latter but not in its blood samples. This may indicate a non-specific, transient infection. Such a limited overlap in trypanosome fauna between these closely related species could be explained by *L. savagei* being particularly attractive to the blood-feeding midges of the genus *Corethrella*. These dipterans are also known to feed on certain hylid frogs [44], and accordingly, two haplotypes from *L. savagei* were also found in *S. ruber* (electronic supplementary material, table S8).

## 4. Conclusions

The diversity and distribution of anuran trypanosomes appear to be primarily shaped by host ecology, the specificity of both vertebrate hosts and invertebrate vectors, as well as vector behaviour. Here, we demonstrate a strong association of the ‘Frog 2’ clade with treefrogs, suggesting that their arboreal lifestyle may drive this pattern. Such an association could result from the preferential feeding of unidentified dipteran vectors in the forest canopy, which maintain a distinct cohort of trypanosome species separate from those infecting understorey anurans. Although host phylogenetic relatedness, and thus similar physiological traits to which trypanosomes may adapt, cannot be ruled out, our findings provide evidence that closely related syntopic frog species do not necessarily share trypanosome communities.

The feeding preferences of vectors may depend not only on ecological niches but also on other aspects of host biology. For example, some haematophagous dipterans, such as the frog-biting midges (Corethrellidae) and the mosquitoes *Uranotaenia lowii* and *Culex territans*, are attracted to male frog mating calls [70–73]. This behaviour may result in lower trypanosome prevalence in female frogs, although infection remains possible, as insects may switch hosts during amplexus [57].

The two well-defined ecological groups of anurans in our dataset harboured comparable trypanosome richness, with 17 species restricted to the arboreal, 19 to the understorey frogs, and three additional species shared between them. The total number of identified trypanosomes was twice that of the examined host species. Considering that (i) our dataset covers only approximately 10% of the anuran diversity in Panama, (ii) sample sizes for most host species are small, and (iii) the number of detected trypanosome species strongly correlates with the host sampling effort, we conservatively estimate that over 300 anuran trypanosome species may occur in Panama alone. The absence of sequence matches with multiple previously published trypanosome sequences from other Neotropical anurans suggests an immense diversity of frog trypanosomes in this region, driven not only by hosts and vectors but also by geographic isolation.

The trypanosomes investigated in this study exhibited substantial variation in host specificity. For instance, two closely related haplotypes (PAF-11 and PAF-12) were confined to *C. fitzingeri*, whereas haplotypes PAF-05, PAF-10, and PAF-13 were detected across members of four anuran families. Another small lineage, encompassing haplotypes PAF-33 and PAF-34, was documented in three families, including Hylidae, which is both phylogenetically and ecologically distinct from the others. Interestingly, these two haplotypes were associated with the ‘hook’ morphotype, previously reported in *T. ogawai* from the fire-bellied newt *C. pyrrhogaster* [60], suggesting that flagellates of this lineage infect diverse and unrelated hosts. Even more striking host switches have been reported for *T. therezieni* and *T. tokoloshi*, belonging to the ‘Frog 1’ and ‘Frog 2’ clades, respectively (figure 3), which have adapted to lizard hosts [23,74].

Vectors have been identified for only a few anuran trypanosomes, limiting our ability to assess their specificity to particular groups of blood-feeding invertebrates. A notable exception is the clade of piscine trypanosomes, which evolved from ancestors infecting anurans and are transmitted exclusively by leeches [1]. Leeches have also been implicated in the transmission of some anuran trypanosomes, including *T. inopinatum*, *T. leptodactyli* and *T. pipientis* [5]. However, the phylogenetic positions of these species remain unknown, as they were studied before the molecular era and no cultures are currently available. The sole exception is *T. fallisi*, a leech-transmitted parasite of toads described in Canada [16,61], which belongs to the ‘Frog 1’ clade (figure 1).

Three groups of blood-feeding dipterans—phlebotomine sandflies, biting midges and mosquitoes—have been implicated in the transmission of anuran trypanosomes [5,57,75]. Sandflies are the most frequently reported vectors of trypanosomes from the ‘Frog 1’ to ‘Frog 3’ clades [21,76], while the frog-biting midges (*Corethrella* spp.) transmit *T. tungarae* of the ‘Frog 4’ clade [57]. Although the vectors of trypanosomes detected in this study remain unidentified, in some cases, we can narrow down the list of candidates. For example, the fully terrestrial lifestyle of *C. fitzingeri* and *P. gaigei*, which lack an aquatic developmental phase (electronic supplementary material, table S10), suggests that the 17 trypanosome haplotypes they host are transmitted by dipterans (electronic supplementary material, table S6). Similarly, *D. auratus* and *S. sila*, which raise their offspring in small, ephemeral pools (electronic supplementary material, table S10), are unlikely to encounter leeches, suggesting that additional 10 trypanosome species are also dipteran-transmitted.

Our data highlight the remarkably high diversity of amphibian trypanosomes and their complex, intertwined patterns of host specificity and geographic distribution. These patterns arise in highly heterogeneous environments, where trypanosomes circulate among taxonomically and ecologically diverse communities of amphibian hosts and invertebrate vectors. However, this extraordinarily rich world is under significant threat, as amphibians are the most endangered and rapidly declining group of vertebrates globally, primarily due to climate change, habitat loss, and emerging infectious diseases [77,78]. Studying this fascinating megadiversity is urgent—before it is too late.

## Data Availability

The data used for the analyses performed in this study are included in the manuscript and its supplementary information or are publicly available (in the case of sequences, see electronic supplementary material, table S2). Supplementary material is available online [79].
